# Diffusion Tensor Tractography Studies on Injured Anterior Cingulum Recovery Mechanisms: A Mini-Review

**DOI:** 10.3389/fneur.2018.01073

**Published:** 2018-12-07

**Authors:** Sung Ho Jang, Jeong Pyo Seo

**Affiliations:** Department of Physical Medicine and Rehabilitation, College of Medicine, Yeungnam University, Daegu, South Korea

**Keywords:** diffusion tensor imaging, diffusion tensor tractography, cingulum, recovery mechanism, brain injury

## Abstract

The cingulum, a major structure in the limbic system, contains the medial cholinergic pathway, which originates from the basalis nucleus of Meynert (Ch 4) in the basal forebrain. The cingulum is involved in various cognitive functions, including memory, attention, learning, motivation, emotion, and pain perception. In this mini-review, 10 studies reporting on recovery mechanisms of injured cinguli in patients with brain injury were reviewed. The recovery mechanisms of the injured anterior cinguli reported in those 10 studies are classified as follows: Mechanism 1, recovery via the normal pathway of the cingulum between the injured cingulum and Ch 4; mechanism 2, recovery through the neural tract between the injured cingulum and the brainstem cholinergic nuclei; mechanism 3, recovery via the lateral cholinergic pathway between the injured cingulum and the white matter of the temporo-occipital lobes; mechanism 4, recovery through the neural tract between the contralesional basal forebrain and the ipsilesional basal forebrain via the genu of the corpus callosum; and mechanism 5, recovery through the neural tract between the injured cingulum and Ch 4 via an aberrant pathway. Elucidation of the recovery mechanisms of injured anterior cinguli might be useful for neurorehabilitation of patients with anterior cingulum injuries. Diffusion tensor tractography appears to be useful in the detection of recovery mechanisms of injured anterior cinguli in patients with brain injury. However, studies on cingulum injury recovery mechanisms are still in the early stages because most of the above studies are case reports confined to a few brain pathologies. Therefore, further studies involving large numbers of subjects with various brain pathologies should be encouraged. In addition, studies on the influencing factors and clinical outcomes associated with each recovery mechanism are warranted.

## Introduction

There are several cholinergic nuclei in the human brain (1–4). Four cholinergic nuclei are located in the basal forebrain and septal region (the medial septal nucleus [Ch 1], the vertical nucleus of the diagonal band [Ch 2], the horizontal limb of the diagonal band [Ch 3], and the nucleus basalis of Meynert [Ch 4]), three in the brainstem (the pedunculopontine nucleus [Ch 5], the laterodorsal tegmental nucleus [Ch 6], and the parabigeminal nucleus [Ch 8]), and one in the thalamus (the medial habenular nucleus [Ch 7]) (2, 3). Ch 4 provides the major cholinergic projections to the cerebral cortex and hippocampus, and the pontine cholinergic system acts mainly through thalamic intralaminar nuclei and provides only minor innervation of the cortex (5, 6). In addition, cholinergic neurons (Ch 1 and Ch 2) in the medial septum innervate mostly the hippocampus, while those of the vertical diagonal band (Ch 3) project to the anterior cingulate cortex (5, 6). As a result, the brain cholinergic system has roles in cortical activity, the sleep-wake cycle, modulating cognitive function, and cortical plasticity, and pathology of the brain cholinergic system can lead to cognitive impairment, age-related cognitive decline, and Alzheimer's disease (4, 7–9). Thus, the cholinergic system of the human brain is important in cognition, especially memory (10).

The cholinergic system of the cerebral cortex is innervated by the medial and lateral cholinergic pathways which mainly originate from Ch 4 (1). After originating from Ch 4, the medial cholinergic pathway joins the white matter of the gyrus rectus and then curves around the rostrum of the corpus callosum to enter the cingulum (1). It supplies cholinergic innervation to the parolfactory, cingulate, paracingulate, and retrosplenial cortices. The lateral cholinergic pathway innervates the remaining portion of the fronto-parieto-temporal cortex (1). As a result, the cingulum is involved in various cognitive functions, especially memory, and an injury of the cingulum could interfere with the corticopetal flow of cholinergic pathways to the above cortical areas (1, 11, 12).

Elucidation of recovery mechanisms following brain injury is important in neurorehabilitation because such information provides a scientific basis for developing neurorehabilitation strategies and predicting prognosis. The recovery mechanisms of the injured brain are based on the following classical concepts for brain plasticity: (1) unmasking of reserve axons and synapses for particular functions following injury of the ordinarily dominant system, and (2) collateral sprouting from an intact neuron to a denervated region (13–15). Recently, the recovery mechanisms of an injured neural tract have been elucidated in more detail; for example, the recovery mechanisms of the corticospinal tract, which have been actively researched, include recoveries through restoration of a normal pathway, perilesional reorganization, and recovery via a collateral pathway or transcallosal or transpontine pathways (16–19). However, relatively little has been reported about the recovery mechanisms of other neural tracts.

Research on the recovery mechanisms of injured cinguli has been limited because identification of the cingulum by using conventional brain magnetic resonance imaging has been impossible because it cannot discern the cingulum from other adjacent structures (20). However, the recently introduced diffusion tensor tractography (DTT) method, which is a derivation of diffusion tensor imaging (DTI), has enabled three-dimensional reconstruction and estimation of the cingulum (21). Several studies have used DTT to describe the recovery mechanisms of injured anterior cinguli in patients with brain injury (20, 22–30).

**Figure 1:**
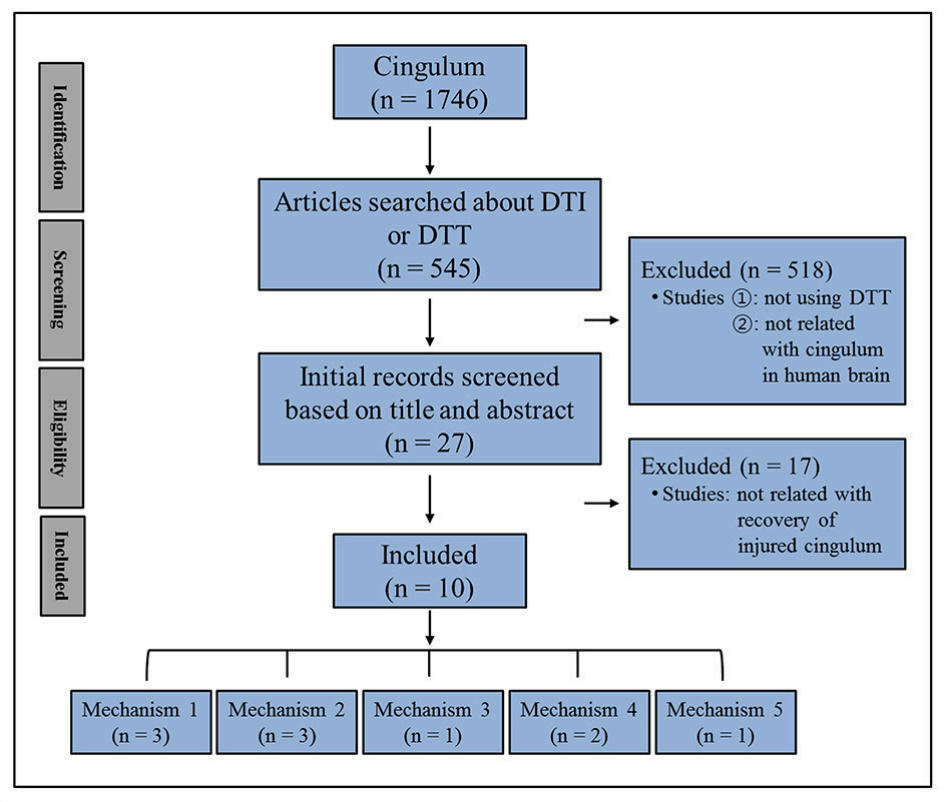
Flow diagram of the approach used to select the studies to be reviewed.

In this mini-review, DTT studies reporting on injured cingulum recovery mechanisms in patients with brain injury are reviewed. Relevant studies in the period 1990 to 2018 were identified by searching within the PubMed, Google Scholar, and MEDLINE electronic databases. The following keywords/abbreviations were used: DTI, DTT, cingulum, anterior cingulum, memory, traumatic brain injury (TBI), stroke, intracerebral hemorrhage (ICH), cerebral infarct, hypoxic-ischemic brain injury (HI-BI), brain tumor, brain injury, brain plasticity, and recovery mechanism. This review was limited to studies of humans with brain injury. We selected the relevant studies according to the flow diagram presented in Figure 1. As a result, ten studies were selected and reviewed (20, 22–30).

## Recovery Mechanisms of an Injured Anterior Cingulum

**Figure 2:**
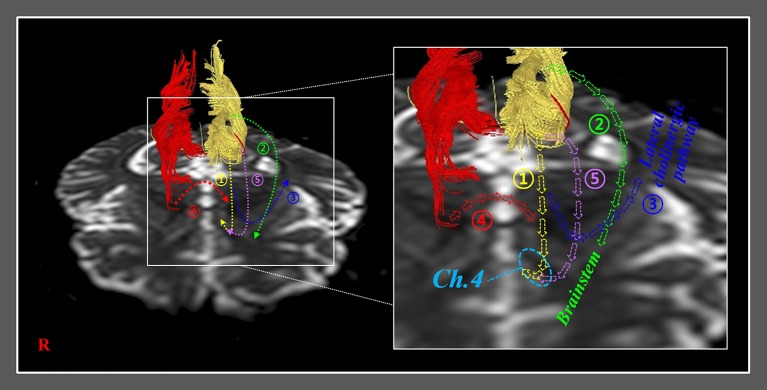
Previously reported recovery mechanisms associated with injured anterior cinguli. Mechanism 1: recovery via the normal pathway of the cingulum between an injured cingulum and the basalis nucleus of Meynert (Ch 4). Mechanism 2: recovery through a neural tract between an injured cingulum and brainstem cholinergic nuclei. Mechanism 3: recovery through a lateral cholinergic pathway between an injured cingulum and the white matter of the temporo-occipital lobes. Mechanism 4: recovery through a neural tract between the contralesional basal forebrain and the ipsilesional basal forebrain via the genu of the corpus callosum. Mechanism 5: recovery through a neural tract between an injured cingulum and Ch 4 via an aberrant pathway.

The recovery mechanisms of the injured anterior cinguli reported in the ten reviewed studies are classified as follows: Mechanism 1, recovery via the normal pathway of the cingulum between an injured cingulum and Ch 4; mechanism 2, recovery through the neural tract between the injured cingulum and the brainstem cholinergic nuclei; mechanism 3, recovery through the lateral cholinergic pathway between the injured cingulum and the white matter of the temporo-occipital lobes; mechanism 4, recovery through the neural tract between the contralesional basal forebrain and the ipsilesional basal forebrain via the genu of the corpus callosum; and mechanism 5, recovery through the neural tract between the injured cingulum and Ch 4 via an aberrant pathway (Figure 2, Table 1) (20, 22–30).

**Table 1:** Previous diffusion tensor imaging studies on the recovery mechanisms of injured cinguli.

**Authors**	**Publication year**	**Number of patients**	**Clinical evaluation method**	**Duration to DTI**	**Pathology of brain injury**	**Recovery mechanism**
Yeo et al. (22)	2012	1	MMSE WAIS	6 months	TBI	2
Seo and Jang (20)	2013	1	MMSE WAIS MAS	7 days 14 months	Hypoxic-ischemic brain injury	1
Seo and Jang (23)	2014	1	WAIS MAS	1 month 7 months	SAH	2
Yoo et al. (24)	2014	20	WAIS MAS	Average 6.98 months	TBI	2
Jang et al (25)	2015	1	WAIS MAS	2 weeks 6 months	TBI	4
Jang et al. (26)	2016	1	MMSE WAIS	4 weeks 9 months	TBI	3
Jang and Kwon (27)	2016	1	MMSE	4 months 4 years	Brain tumor, ICH	1
Jang and Seo (28)	2016	1	MMSE WAIS	6 weeks 6 months 10 months	TBI	5
Jang et al. (29)	2018	1	MAS	3 months 2 years	TBI	1
Jang et al. (30)	2018	1	MMSE	3 weeks	ICH IVH SAH	4

*DTI, Diffusion Tensor Imaging; TBI, Traumatic Brain Injury; MMSE, Mini-Mental State Examination; WAIS, Wechsler Adult Intelligence Scale; MAS, Memory Assessment Scale*.

### Mechanism 1: Recovery via the Normal Pathway of the Cingulum

In 2013, Jang and Seo reported on neural recovery of an injured cingulum in a patient who had HI-BI and a subarachnoid hemorrhage (SAH) that was observed on follow-up DTT (20). The patient showed severe cognitive impairment; therefore, the authors were unable to perform an evaluation using the Mini-Mental State Examination (MMSE: full score = 30) or other cognitive function tests (31). At 14 months after onset, the patient's cognition had improved and she scored 24 on the MMSE. Other cognitive function test results were as follows: Total intelligence quotient (IQ) on the Wechsler adult intelligence scale (WAIS: 81); Memory assessment scale (MAS) scores: short-term memory, 88 (21%ile); verbal memory, 62 (1%ile); visual memory, 68 (2%ile); and global memory, 56 (< 1%ile) (32, 33). On DTT at 7 days after brain injury, discontinuations of both cinguli anterior to the genu of the corpus callosum were observed. However, on 14-month follow-up DTT, the right cingulum was observed to have elongated to the right basal forebrain through the normal cingulum pathway. The authors suggested that the recovery of memory impairment in this patient was attributed to the elongation of the right injured cingulum via the normal pathway of the cingulum (20).

In 2016, Jang and Kwon reported on changes to the anterior cingulum in a patient who underwent craniectomy and removal of meningioma and had concurrent ICH (27). The patient showed mild cognitive impairment (MMSE score = 22) (31). By contrast, at 4 years after onset, his cognitive impairment had improved to an MMSE score of 28. The 4-month DTT of the patient showed discontinuations in both anterior cinguli. On 4-year follow-up DTT, the left anterior cingulum was shown to have elongated to the basal forebrain through the normal pathway of the anterior cingulum. As a result, the authors concluded that their observations appeared to indicate recovery of the left injured cingulum, and the reduction in the patient's cognitive impairment was ascribed to the recovery of the left injured cingulum (27).

In 2018, Jang et al. reported on a patient with mild TBI who, on follow-up DTT, showed recovery of an injured cingulum concurrent with improvement of short-term memory (29). The patient showed mild memory impairment at 3 months after onset: MAS scores: global memory, 95 (37%ile); short-term memory, 75 (5%ile); verbal memory, 80 (9%ile); and visual memory, 112 (79%ile) (33). By contrast, 2 years after onset, his mild memory impairment had reduced and his memory scores indicated a normal state: MAS scores: global memory, 104 (61%ile); short-term memory, 95 (37%ile); verbal memory, 101 (53%ile); and visual memory, 106 (66%ile) (33). On 3-month DTT, discontinuation of the right anterior cingulum over the genu of the corpus callosum was observed; however, on 2-year DTT, the discontinued right anterior cingulum was observed to have elongated to the right basal forebrain. As a result, the authors concluded that elongation to the right basal forebrain of the discontinued anterior cingulum appeared to be the recovery mechanism associated with the injured cingulum following a mild TBI (29).

### Mechanism 2: Recovery Through the Neural Tract Between an Injured Cingulum and the Brainstem Cholinergic Nuclei

In 2012, Yeo et al. presented a case report on a patient who had experienced a traffic accident and underwent conservative management for SAH in the right frontal and left Sylvian fissure (22). The patient revealed severe cognitive impairment (MMSE score = 15) (31). In contrast, at a 6-month post-onset cognitive evaluation, there was a reduction in cognitive impairment (MMSE score = 24 and WAIS score = 83) (31, 32). Moreover, on 6-month DTT of the patient, they observed discontinuations of both cinguli above the genu of the corpus callosum; in contrast, the left cingulum was observed to be connected to the left Ch 5 in the brainstem via a neural tract that passed through the anterior corona radiata and the thalamus. As a result, the authors assumed that the cognitive improvement in this patient was related to the recovery of the neural connection between the injured cingulum and Ch 5 in the brainstem (22).

In 2014, Seo and Jang reported on a patient who showed unusual neural connections between the anterior portions of injured cinguli and the brainstem cholinergic nuclei following SAH (23). The patient showed memory impairment at 5 weeks after onset but his memory function showed improvement to within the normal range at 7 months after onset: His total IQ on WAIS increased from 104 to 125; moreover, there were widespread improvements in MAS scores: global memory, from 79 (8%ile) to 104 (61%ile); short-term memory, from 111 (77%ile) to 114 (83%ile); verbal memory, from 94 (35%ile) to 111 (77%ile); and visual memory, from 71 (3%ile) to 97 (42%ile) (32, 33). On both 1- and 7-month DTT of the patient, discontinuations of both cinguli above the genu of the corpus callosum were observed. On the 1-month DTT, both cinguli were connected to their respective bilateral Ch 5 via neural tracts that passed through the thalamus; in contrast, on the 7-month DTT, the left neural tract was not visible, and the right neural tract was connected to the right Ch 6. The authors suggested that this unusual neural connection between the anterior portions of injured cinguli and the brainstem cholinergic nuclei was the recovery mechanism associated with cholinergic innervation of the injured cinguli (23).

During the same year (2014), Yoo et al. investigated the relationship between cognition and the neural connections from injured cinguli to brainstem cholinergic nuclei in patients with TBI (24). Twenty chronic TBI patients who showed discontinuation between both anterior cinguli and the basal forebrain on DTT were recruited for the study. Eight patients who had neural connections between their injured cinguli and various brainstem cholinergic nuclei (Ch 5, Ch 6, and Ch 8) had better MAS short-term memory scores than 12 patients who did not have such connections. The authors concluded that the observed neural connections between the injured cinguli and the brainstem cholinergic nuclei appeared to be a recovery mechanism of injured cinguli (24).

### Mechanism 3: Recovery Through the Lateral Cholinergic Pathway Between an Injured Cingulum and the White Matter of the Temporo-Occipital Lobes

In 2016, Jang et al. reported on a patient with TBI who showed recovery of an injured cingulum that progressed via the lateral cholinergic pathway (26). At 4 weeks after TBI onset, the patient exhibited mild cognitive impairment; however, cognition had improved at the 9-month evaluation (MMSE from 21 to 29; total IQ from 85 to 96; verbal IQ from 86 to 96; performance IQ from 84 to 97; verbal immediate recall from 26.76 to 56.75%ile; visual immediate recall from 29.81 to 89.49%ile; verbal delayed recall from 24.51 to 78.23%ile; visual delayed recall from 11.70 to 89.07%ile; verbal recognition from 43.25 to 85.31%ile; and visual recognition from 0.94 to 93.06%ile) (31–33). On 4-week DTT, discontinuations in both cinguli were observed superior to the genu of the corpus callosum. However, on 9-month DTT, the discontinued anterior part of the right cingulum was observed to have elongated inferoposteriorly via an unusual neural tract that passed through the external capsule and the white matter of the temporo-occipital lobes. The authors concluded that recovery of an injured cingulum via a lateral cholinergic pathway is a recovery mechanism of an injured cingulum (26).

### Mechanism 4: Recovery Through a Neural Tract Between the Contralesional Basal Forebrain and the Ipsilesional Basal Forebrain via the Genu of the Corpus Callosum

In 2015, Jang et al. reported on changes in DTT results for the cingulum that coincided with changes in cognitive impairment in a patient with TBI (25). Upon evaluation of cognitive function performed 2 weeks after onset, the patient revealed severe cognitive impairment; however, cognition was improved at the 6-month evaluation. Improvements include total IQ from 65 to 82; MAS global memory from 61 (1%ile) to 102 (55%ile); and MAS immediate memory from 83 (13%ile) to 107 (68%ile) (32, 33). On DTT 2 weeks after onset, the authors observed discontinuations in both cinguli anterior to the genu of the corpus callosum. However, on a 6-month follow-up DTT, the left cingulum had elongated to the left basal forebrain and the right cingulum was connected to left basal forebrain by a new tract that passed anterior to the genu of the corpus callosum. That new tract was not observed on the DTT results obtained at 2 weeks after onset (25).

Recently, Jang et al. reported on a patient who had developed new neural tracts between the injured anterior cinguli and the basal forebrain following ICH, intraventricular hemorrhage, and SAH after the rupture of an aneurysm in the left middle cerebral artery (30). When beginning rehabilitation at 3 weeks after onset, the patient showed severe cognitive impairment (MMSE: uncheckable) (31). At that time, DTT revealed the discontinued right anterior cingulum was connected to the left basal forebrain via the genu of the corpus callosum. In addition, the discontinued left anterior cingulum was shown to be connected via an unusual neural tract from the right anterior cingulum to the left basal forebrain. The authors suggested that development of this unusual neural tract between the basal forebrain and the injured cinguli via the genu of the corpus callosum, after interruption of cholinergic innervation from the basal forebrain by complete injury of the anterior cingulum, might have been the result of reorganization of cholinergic innervations following the stroke (30).

### Mechanism 5: Recovery Through a Neural Tract Between an Injured Cingulum and the Basalis Nucleus of Meynert via an Aberrant Pathway

In 2016, Jang et al. reported on a patient who, following TBI, appeared to show recovery of an injured anterior cingulum via an aberrant neural tract between an injured cingulum and Ch 4 (28). The patient showed improvement of cognitive function on the MMSE with scores of 10 at 2 months, 13 at 6 months, and 26 at 10 months after onset (31). Total IQ was 90 on the WAIS at 10 months after onset (32). DTT at 6 weeks after onset showed discontinuation superior to the genu of the corpus callosum in both cinguli. However, on a 6-month DTT, the discontinued anterior part of the right cingulum was shown to have elongated anteriorly through the anterolateral subcortical white matter of the cingulum. On 10-month DTT, this elongated neural tract of the right cingulum was connected to the right Ch 4 in the basal forebrain. The authors concluded that the aberrant neural tract between the injured anterior cingulum and Ch 4 appeared to be a recovery mechanism of an injured cingulum (28).

## Conclusions

In this mini-review article, 10 studies (six TBI studies; three stroke studies; and one HI-BI study) reporting recovery mechanisms of injured cinguli in patients with brain injuries were reviewed (20, 22–30). The frequencies of occurrence of the five different recovery mechanisms of injured cinguli are: Mechanism 1, 3 papers; mechanism 2, 3 papers; mechanism 4, 2 papers; mechanism 3, 1 paper; and mechanism 5, 1 paper. Based on the presence of multiple reports describing mechanisms 1 and 2, recovery mechanisms through the normal pathway of the anterior cingulum and the neural tract between an injured cingulum and the brainstem cholinergic nuclei appeared to be reliable. However, the other recovery mechanisms are less assured because of the shortage of published reports on those mechanisms.

Based on the observations in the reviewed papers, it appears that injured cingulum recovery mechanisms are based on reorganization of the cholinergic innervation between the injured cingulum and the cholinergic nuclei as a means to obtain cholinergic innervation after the loss of cholinergic innervation between the basal forebrain and the anterior cingulum (1, 34–36). Studies of injured anterior cinguli that describe recovery mechanisms might be useful in the neurorehabilitation of patients with anterior cingulum injury; for example, neuromodulation such as that provided by repetitive transcranial magnetic stimulation or transcranial direct current stimulation, which have been popularly applied in neurorehabilitation in recent years, can be applied to facilitate or induce a possible recovery mechanism of injured cinguli in patients with brain injuries (37–40). In addition, the information provided in those papers suggests that DTT appears to be useful in the detection of the particular recovery mechanism associated with injured anterior cinguli in a patient with brain injury. However, studies on this topic are uncommon, and research into such recovery mechanisms is still in the early, descriptive stage because most of the reviewed studies were case reports and were confined to a few brain pathologies including TBI, stroke, and HI-BI. Therefore, further studies involving large numbers of subjects and a wider variety of brain pathologies should be encouraged. In addition, studies into the influencing factors and clinical outcomes associated with each recovery mechanism are warranted. Despite the advantages of DTT, the limitations of DTT also need to be considered because three-dimensional reconstruction of brain regions that involve fiber complexity and fiber crossing can prevent DTT from fully reflecting the underlying fiber architecture, resulting in a possible underestimation of the status of the neural tract of interest (41–43).

## Author Contributions

SHJ: study concept and design, manuscript development, and writing. JPS: study concept and design, acquisition and analysis of data, and manuscript authorization.

### Conflict of Interest Statement

The authors declare that the research was conducted in the absence of any commercial or financial relationships that could be construed as a potential conflict of interest.
